# An Event-Based Neurobiological Recognition System with Orientation Detector for Objects in Multiple Orientations

**DOI:** 10.3389/fnins.2016.00498

**Published:** 2016-11-04

**Authors:** Hanyu Wang, Jiangtao Xu, Zhiyuan Gao, Chengye Lu, Suying Yao, Jianguo Ma

**Affiliations:** School of Electronic Information Engineering, Tianjin UniversityTianjin, China

**Keywords:** address-event representation (AER), visual tracking, dynamic vision senses (DVS), MNIST, spiking neural network (SNN), multi-orientation object recognition

## Abstract

A new multiple orientation event-based neurobiological recognition system is proposed by integrating recognition and tracking function in this paper, which is used for asynchronous address-event representation (AER) image sensors. The characteristic of this system has been enriched to recognize the objects in multiple orientations with only training samples moving in a single orientation. The system extracts multi-scale and multi-orientation line features inspired by models of the primate visual cortex. An orientation detector based on modified Gaussian blob tracking algorithm is introduced for object tracking and orientation detection. The orientation detector and feature extraction block work in simultaneous mode, without any increase in categorization time. An addresses lookup table (addresses LUT) is also presented to adjust the feature maps by addresses mapping and reordering, and they are categorized in the trained spiking neural network. This recognition system is evaluated with the MNIST dataset which have played important roles in the development of computer vision, and the accuracy is increased owing to the use of both ON and OFF events. AER data acquired by a dynamic vision senses (DVS) are also tested on the system, such as moving digits, pokers, and vehicles. The experimental results show that the proposed system can realize event-based multi-orientation recognition. The work presented in this paper makes a number of contributions to the event-based vision processing system for multi-orientation object recognition. It develops a new tracking-recognition architecture to feedforward categorization system and an address reorder approach to classify multi-orientation objects using event-based data. It provides a new way to recognize multiple orientation objects with only samples in single orientation.

## Introduction

Visual object recognition is useful in many applications, such as vehicle recognition, face recognition, digit recognition, posture recognition, and fingerprint recognition. Most object recognition techniques depend on capturing and processing sequences of still frames, which limits algorithmic efficiency when dealing with fast-moving objects. If precise object recognition is required, sequences of computationally demanding operations need to be performed on each acquired frame. The computational power and speed of such tasks make it difficult to achieve real-time autonomous systems (Triesch and Malsburg, 2001; Han and Feng-Gang, 2005). On the other hand, vision sensing and object recognition in brains are performed without using the “frame” concept, but a continuous flow of visual information in the form of temporal spikes instead. Thus, less information is required to identify objects, which improves recognition efficiency. Recent years have witnessed accelerative efforts in biomimetic visual sensory system for object recognition and tracking.

Biomimetic visual sensory system based on address-event representation (AER) was proposed by Mead's Lab at California Institute of Technology (Mahowald and Mead, 1991). The AER vision sensor is a novel type of vision devices like biological retinas adopting event-based information encoding and data communication. Notable examples of such biomimetic vision sensors include the earliest examples of the spiking silicon retina (Culurciello et al., 2003; Zaghloul and Boahen, 2004), the more recent Dynamic Vision Sensor (DVS; Lichtsteiner et al., 2008; Serrano-Gotarredona and Linares-Barranco, 2013), asynchronous time-based image sensor (ATIS; Posch et al., 2008, 2011), and the DAVIS sensor (Brandli et al., 2014). An AER vision sensor contains a pixel array, where each pixel can individually monitor the relative change of light intensity and output an event if the change exceeds a user-defined threshold. Events are asynchronously encoded in x, y coordinates resembling the precisely timed electrical impulses or spikes of the spatially arranged optical nerves stemming from the retina to the primary visual cortex (Serre et al., 2007). An asynchronous row and column arbitration tree circuits can process the pixel requests and arrange the output sequence in a fairly random manner when multiple pixels request to output events at the same time (Boahen, 2000; Aung Myat Thu et al., 2011). Only one request is granted at a time. Because only relative change events are output, biomimetic vision sensors have the advantage of asynchronous, high temporal resolution, and sparse representation of the scene.

With the emergence of these asynchronous vision sensors, many studies focusing on event processing were reported. Hence, many models and variants such as the Neocognitron (Fukushima, 1980), convolutional neural network (CNN; Lecun et al., 1998), and Hierarchical model and X (HMAX; Riesenhuber and Poggio, 1999) are introduced to extract features in a variety of object recognition tasks. In 2008, Event-driven convolution chips for neuromorphic spike-based cortical processing have been designed for feature extraction using programmable kernels (Serrano-Gotarredona et al., 2008). In addition, the convolution chips were combined with other AER processing blocks, such as neural networks, to build larger biomimetic visual sensory system for classification (Serrano-Gotarredona et al., 2009; Perezcarrasco et al., 2013). In recent years, the network in the biomimetic visual sensory system for classification has been improved. An algorithm for the size and position invariant categorization of objects, especially for human postures has been exploited in real-time video sequences from the address-event temporal-difference image sensors (Chen et al., 2012). Two years later, an event-driven feedforward categorization system has been proposed, where a “tempotron” classifier is adopted and a motion symbol detection module is added to capture motion symbols (Zhao et al., 2015). A hierarchical spiking neural network model called HFirst has been developed for object recognition (Orchard et al., 2015b). Besides, a biologically-inspired Gabor feature approach based on spiking neural networks with Leaky-Integrate and Fire neurons has been presented (Tsitiridis et al., 2015). A Synaptic Kernel Inverse Method (SKIM) based on principles of dendritic computation is applied to N-MNIST dataset (Orchard et al., 2015a) to perform a large-scale classification task (Cohen et al., 2016).

Despite the success of these methods, it is still challenging to fully exploit the advantage of AER. The majority of these event-based categorization systems can only recognize the objects moving in a specified orientation which limits their application for classification. If the target object moves in free angles, these systems may fail in identification. In order to recognize the identical objects moving in different orientations, a large amount of multi-orientation samples should be introduced into the existing event-based categorization systems as training samples, which increases the sample acquisition time and the training time significantly. Therefore, we develop a new system architecture introducing a tracking mechanism into the visual sensory system in this paper to solve this problem. In fact, several tracking algorithms have been developed for AER visual sensor. An event clustering algorithm is introduced for traffic monitoring, where clusters can change in size, but the shape of clusters is restricted to a circular form (Litzenberger et al., 2006a,b). In addition, a fast sensory motor system using a cluster tracker algorithm has been built to demonstrate dynamic vision sensor's high temporal resolution properties in Delbrück and Lichtsteiner (2007). Several event-based tracking algorithms can also be found at (Delbrück, 2006). They have been recently applied to track particles in microrobotics (Ni et al., 2012) and in fluid mechanics (Drazen et al., 2011). Recently, an asynchronous event-based multiple kernels algorithm for tracking is presented and it features high stability and feasibility (Lagorce et al., 2015).

In this paper, a multiple orientation event-based recognition system is presented based on a modified Gaussian blob tracking algorithm (Lagorce et al., 2015) and the event-driven feedforward categorization system (Zhao et al., 2015). In this system, the ON and OFF events, two different polarity events, transmitted by the AER vision sensor are separately treated to enhance the difference of each extract feature type, which can improve the efficiency of target recognition compared to existing event-based categorization systems. Only regular samples with single orientation are adopted for training. In the test process, an orientation detector is introduced to judge the moving orientation of the target object, and its extracted feature maps are adjusted according to its orientation and addresses LUT before feeding to the classifier. These operations enrich the system function recognizing objects moving in different orientation along its positive direction, with training samples only in one orientation.

The rest of this paper is structured as follows. The next section describes the system architecture overview as well as its building blocks. The experimental results are reported in Section Results. The main application fields, advantages and limitations of this system are discussed in Section Discussion.

## Materials and methods

In the present event-based object recognition systems, a biomimetic frame-free vision sensor named DVS is used to acquire image data, and then the data is transmitted into bio-inspired feature extraction module. In the bio-inspired feature extractor, each event is sent in parallel to a battery of orientation filters based on the Gabor functions, and convolution operation is performed on the fly. After that, a maximum (MAX) operation (Zhao and Chen, 2011) is applied to select the maximal response within its receptive field. A spiking neural network (SNN; Wulfram and Werner, 2002) is used as a classifier receiving all the peak responses with time and address information to train the weights of each address, and then all the weights are stored into a weights lookup table (weights LUT). In the process of testing, SNN invokes them for pattern recognition.

**Figure 1:**
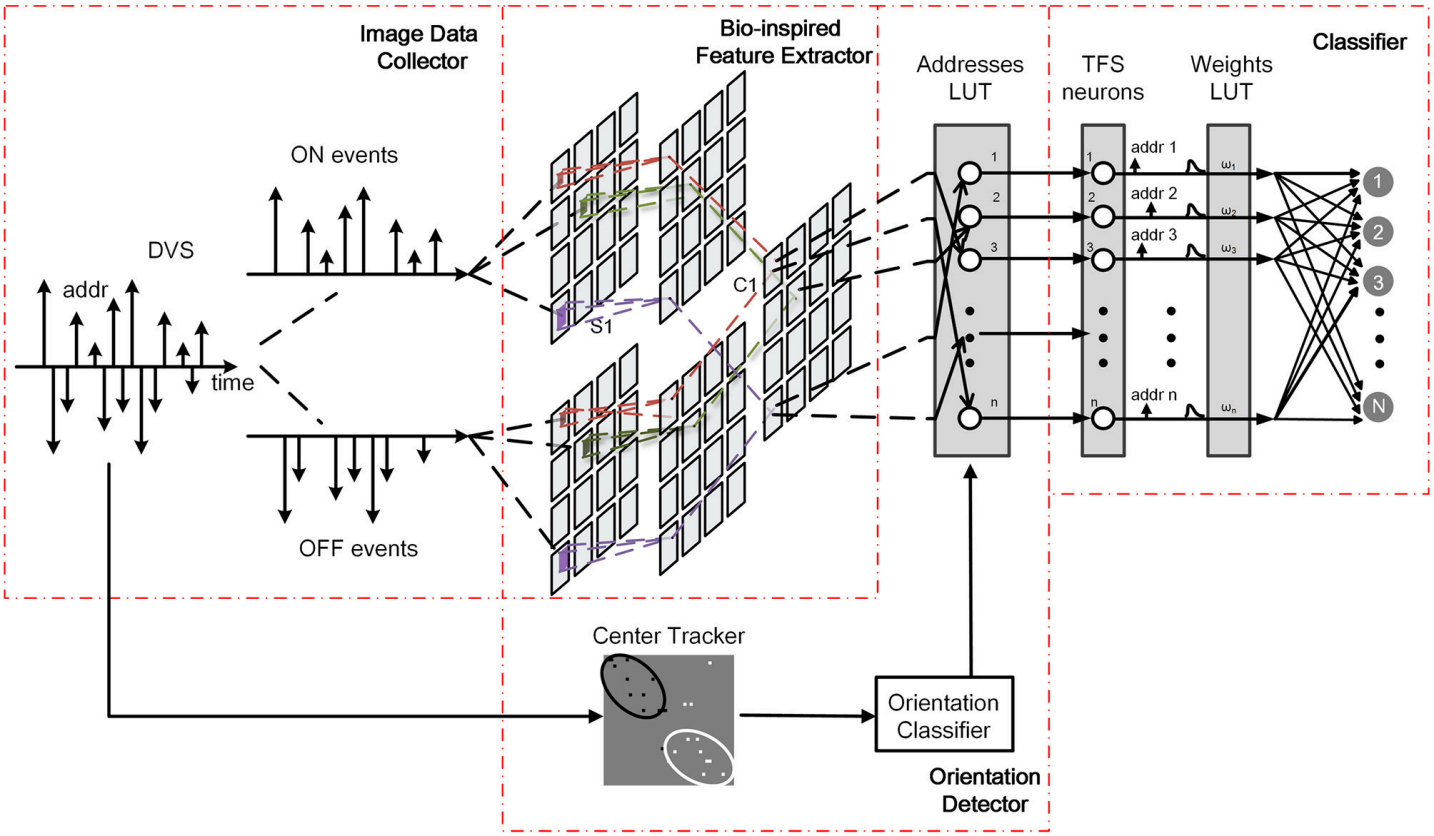
**Architecture of the proposed system**.

In the proposed system, an orientation detector is introduced. The architecture is illustrated in Figure 1. It consists of three modules, namely a bio-inspired feature extractor, an orientation detector, and a classifier. The bio-inspired feature extractor is used to extract the orientation and scale features. The orientation detector aims to find the orientation of the moving target. The classifier, as the name implies, determines the classification of each detected object.

The workflow of the new proposed system is the same as the present system mentioned above in the process of training. In the testing process, AER data is transmitted into bio-inspired feature extraction module and orientation detection module simultaneously. Each event successively accesses to the modified Gaussian blob tracker to calculate the center positions of ON and OFF events, and then the orientation of motion is estimated. Moreover, the orientation detector adjusts addresses of the peak responses generated by bio-inspired feature extraction module according to the orientation. Spiking neural network receives all the peak responses with new addresses for pattern recognition.

### Features extraction

The bio-inspired feature extraction block as the foremost part is a hierarchical architecture inspired by a recent model of object categorization in the primate visual cortex (Serre et al., 2007). To simplify the calculation and ensure the precision, architecture with two layers is adopted to extract line feature information from the input data. The hierarchical architecture of multiple feature extraction can be divided into three parts that are event-driven convolution, forgetting mechanism, and MAX operation. The overall data flow can be summarized as follows: each input event acquired by DVS is fed into a group of simple filters named “S1,” where the event-driven convolution (Chen et al., 2004; Serre, 2006) and forgetting mechanism (Zhao and Chen, 2011) are accomplished in this layer. Then the results in “S1” access to the second layer “C1” (Complex Cells) for maximum (MAX) operation. Thus, the peak responses are extracted after “C1.” The detailed operation is described as follows.

**Figure 2:**
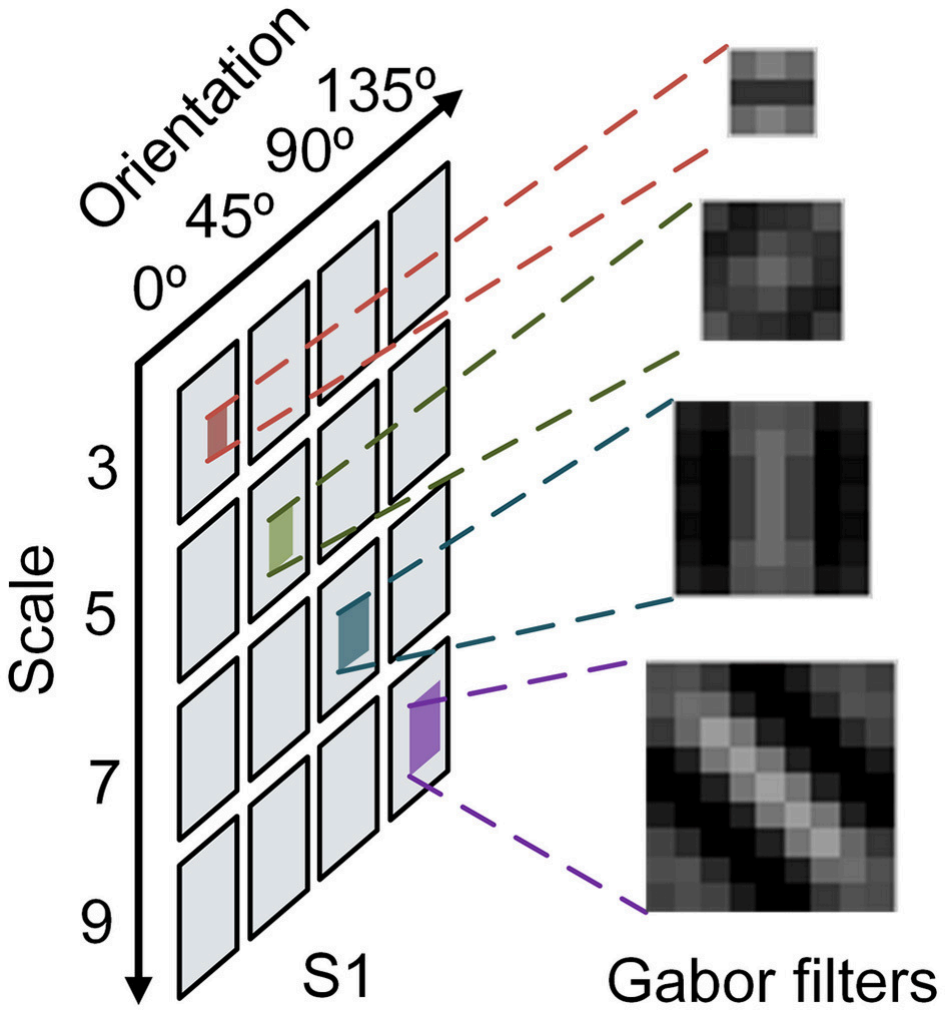
**Convolution kernel in different scales and orientations**.

Once an event accesses “S1” layer, each convolution kernel will be added to the corresponding address of its feature map in parallel. In this paper, convolution kernels are generated by Gabor function, since multiple Gabor feature maps can achieve selectivity in space, spatial frequency, and orientation (Serre et al., 2007). Sixteen convolution kernels are used to construct a network with four scales (*s* = 3, 5, 7, 9) and four orientations (θ = 0°, 45°, 90°, and 135°) for feature extraction. The four of them are shown in Figure 2. In order to eliminate the interference between two polarities of events, ON events and OFF events should be handled separately, which means that there are 32 feature maps actually. In the meantime, to mitigate the impact of very old motion events on current responses, a forgetting mechanism is used to update event's responses periodically by decay operation. The old event's responses are (decreased or increased) rely on a linear function connected with time interval. When the previous value of a point is positive, the value decreases with one decay operation; and when the previous value is negative, it increases with one decay operation. In summary, the decay operation gradually lessens the absolute value of a point. Finally, if the time interval is long enough, the responses will approach zero. After all the events flow into “S1” layer, all the neurons in multiple feature maps send their responses to “C1” layer.

In the next layer referred as “C1” layer, each complex cell receives the responses from the corresponding feature map in “S1” layer. Only minority neurons can survive in “C1,” because the MAX operation picks out the neighboring maximum responses where the scale of neighborhood is the same as the size of convolution kernel. The MAX operation benefits to find out the center of feature and makes further efforts to filter out the redundancy. At the same time, Threshold comparison works together to avoid the dispensable background noise events in the process of event collection and feature extraction. Only if it is superior to the threshold, the peak response can survive. After the MAX operation and threshold comparison operation, all survived peak responses from ON and OFF events are superposed together according to the corresponding maps, and then they are transmitted to the next block.

### Object categorization

In order to tackle the responses from multiple feature extraction for categorization, we adopt a hierarchical Spiking Neural Network (SNN; Wulfram and Werner, 2002). SNN falls into the third generation of neural network model, increasing the level of realism in a neural simulation. In addition to neuronal and synaptic state, it also incorporates the concept of time into its operating model. Hence, not only the location (address) of event flow can be communicated within the network, but also the generated time is correctly modeled by the digital communication to reflect the spatio-temporal behavior.

In this paper, a simple leaky integrate-and-fire (LIF) neuron (Wulfram and Werner, 2002) combining spike-Timing-dependent plasticity (STDP) rule (Markram et al., 1997) is selected as the neuron model. All the responses from “C1” feature maps need a response-to-spike conversion which is in time-to-first spike (TFS) mode. And then, the spikes achieve a virtually fully connected system associated with an address. Each input spike contributes a postsynaptic potential (PSP) with fast-rising and slow-decaying to a tempotron neuron via its corresponding address (Zhao et al., 2015). With respect to an input spike received at time *t*_*i*_, the normalized PSP kernel *K* is defined as:

(1) $$\begin{array}{r} K\left(t-t_{i}\right)=V_{0}\times \left[exp\left(\frac{-\left(t-t_{i}\right)}{\tau_{\text{m}}}\right)-exp\left(\frac{-\left(t-t_{i}\right)}{\tau_{\text{s}}}\right)\right], \end{array}$$

where τ_m_ and τ_s_ indicate the two decay time constants of membrane integration and synaptic currents, respectively, and τ_s_ is set to be τ_m_/4. The membrane time constant τ_m_ is set as 0.1. *V*_0_ normalizes PSP, so that the maximum value of the kernel is set as 1. In addition, the tempotron neuron's membrane potential is the weighted summation of the PSPs from all the input spikes:

(2) $$\begin{array}{r} V\left(t\right)=\underset{i}{\sum }\omega_{i}\underset{t_{i}}{\sum }K\left(t-t_{i}\right)+V_{\text{rest}}. \end{array}$$

**Figure 3:**
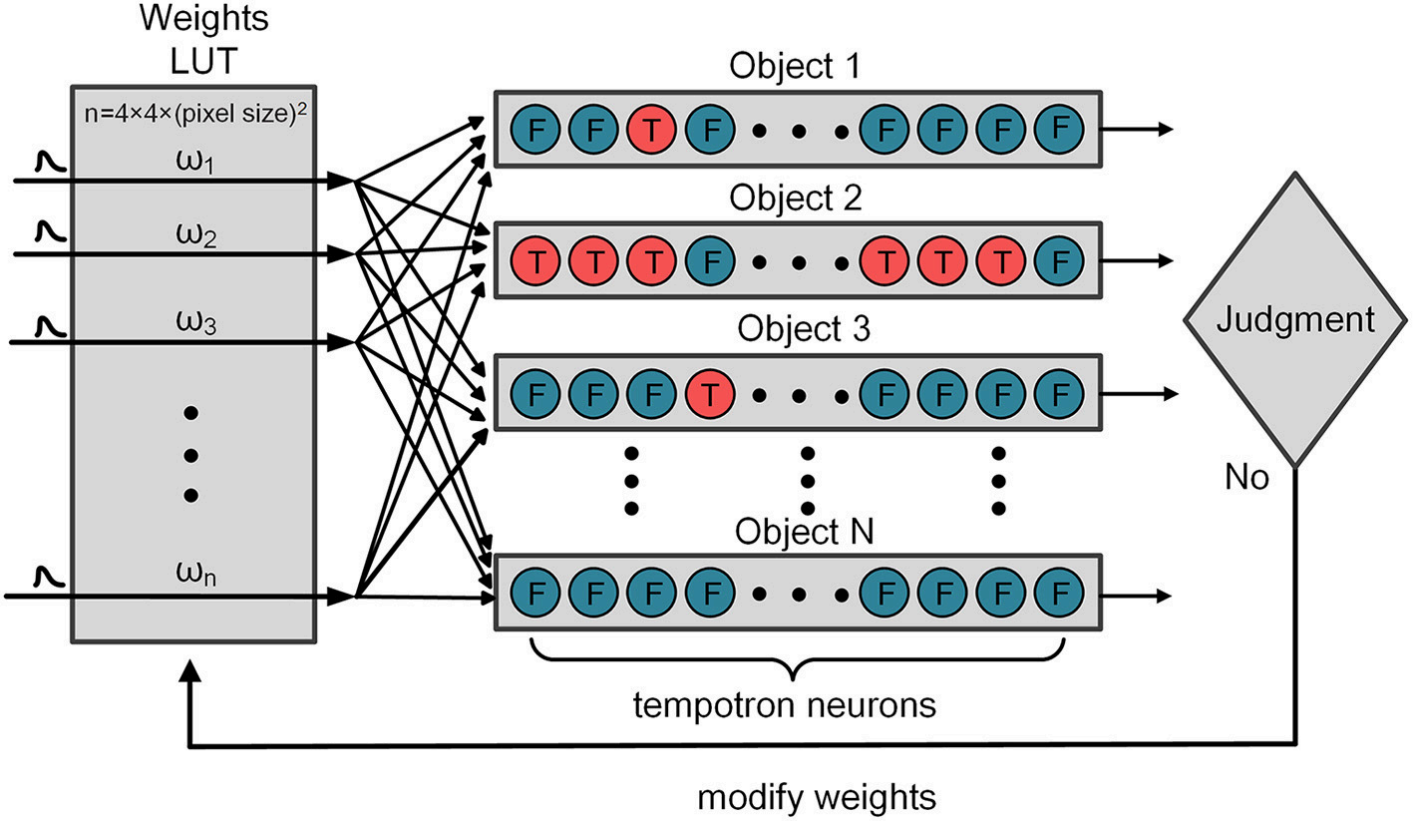
**The procedure of weights modification**.

In equation (2), ω_*i*_ and *t*_*i*_ are the synaptic efficacy (weight) and the firing time of the *i*th afferent synapse, respectively. *V*_rest_ is the resting potential of the neuron. As the input of PSPs increases, the membrane potential of tempotron neuron rises. When the tempotron neuron's membrane potential exceeds a specified threshold, it will fire and trigger the release of further neurotransmitter such as an output spike. After firing, the tempotron neuron shunts all the following input spikes and the potential gradually returns to the resting level. In the training stage, the weights LUT is initialized with random weights, and all the weights will be revised by the tempotron learning rule, as illustrated in Figure 3. According to the known type of classification, the sequence numbers of fire neuron can be acquired. Each tempotron neuron in different categorization has a set of weights and each weight is initialized with a random value. If the tempotron neuron is supposed to fire (or not fire, on the other hand) but it actually fails to do so (or does fire, vice versa), the judgment mechanism will send out a signal to modify the weights. Finally, the optimized weights of address are stored in weights LUT ready for the use of the testing process.

In fact, thanks to the spatio-temporal AER spikes and the MAX operation, only very few neurons survive after competition. Moreover, to reduce computational complexity and improve power efficiency, only a very small subset of “C1” responses (less than 100) are selected as the inputs. These responses can fully represent all the features in different feature maps. This work uses the one-hot coding scheme to label the tempotron neurons. If sample belongs to the first class, then the first tempotron neuron output is labeled 1 (which means it should fire), and all the other neuron outputs are labeled 0 (not fire). This encoding is a simple method to obtain the classification result by checking which neuron fires. In addition, to further improve the performance, multiple neurons are used for each category (Yu et al., 2013). The number of tempotron neurons for each category is set as 10. We then use the majority voting scheme to make the final decision: to check which category has the most firing neurons.

### Center tracking and orientation detection

**Figure 4:**
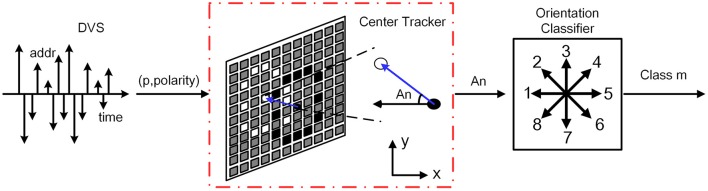
**The procedure of center tracking and orientation detection**. The events first flow into the center tracking unit to calculate the centers of ON and OFF events and obtain the angle *An*. And then the orientation classifier categorizes the angle *An* into the nearest class *m*. *m* represents the number of categories which is from 1 to 8.

The above-mentioned architectures of feature extraction and classification are inspired by a complete event-driven feedforward categorization system (Zhao et al., 2015). However, this architecture is not useful when the detected object moves in multiple orientations. In this paper, an orientation detector is introduced to enable biomimetic event-based recognition system to recognize the same object moving in different orientations along its forward direction. In the training process, the working flow is exactly the same with the previous one. All the training samples are moving along the reference positive orientation, which is defined as the reverse direction of the x axis. In the testing process, the orientation detector works parallelly with the feature extractor. It estimates the centers of ON and OFF events using a modified algorithm based on Gaussian blob tracking (Lagorce et al., 2015). The method of Gaussian blob tracking allows adaptation to the spatial distribution of events by continuously correcting the Gaussian size, orientation, and location with each incoming event, so it is easy to track clouds of events by using bivariate normal distribution. Thus, the orientation of the moving target can be calculated according to the center of ON and OFF events as shown in Figure 4. Moreover, the orientation detection classifies the orientation into 8 classes. Once all the events flow into the orientation detector, a digital label representing the orientation category is transmitted to a prepared addresses lookup table (addresses LUT) as shown in Figure 1.

The main assumption for the evaluation of the event flow is the invariance of light intensity undergoing a small motion over infinitesimally short duration. When an object moves, the pixels generate events which geometrically form an events cloud that represents the spatial distribution of the observed shape. There are two trackers to detect the clouds of ON and OFF events, respectively. Note that if all the events no matter ON or OFF are tracked together in one tracker, the detected result is the center of the object.

We treat the spatial distribution of events cloud as a bivariate normal distribution which is also called bivariate Gaussian distribution (Valeiras et al., 2015). The probability density function generated by the Gaussian distribution around the tracker can be calculated with the incoming events. If the computed result is superior to a predefined threshold, which indicates the position of the current event is close to the tracker, the parameters of tracker should be corrected using a simple weighting strategy that combines the last distribution and the current event information. Therefore, the position and size of a Gaussian tracker are updated.

After all the corrections by the incoming events, we can obtain the detected object's position *p* = (x, y)^T^ and the orientation of motion. The orientation of motion is the vector between the center of OFF events and ON events ($\vec{{\text{p}}_{-}{\text{p}}_{+}}$ or $\vec{{\text{p}}_{\text{+}}{\text{p}}_{-}}$), where the subscripts (“+” and “−”) represent ON and OFF events. The use of $\vec{{\text{p}}_{-}{\text{p}}_{\text{+}}}$ or $\vec{{\text{p}}_{+}{\text{p}}_{-}}$ depends on the light intensity of the object and the background, which should be determined by combining with the deviation between these two events center. Then the deviation angle *An* indicating the motion orientation off the reference positive orientation clockwise can be defined as follows:

(3) $$\begin{array}{l} An=\{\begin{array}{ll} \frac{180}{\pi }\text{arccos}\left(\frac{-\Delta x}{\sqrt{{\left(\Delta x\right)}^{2}+{\left(\Delta y\right)}^{2}}}\right) & \Delta y\geq 0\\ 360-\frac{180}{\pi }\text{arccos}\left(\frac{-\Delta x}{\sqrt{{\left(\Delta x\right)}^{2}+{\left(\Delta y\right)}^{2}}}\right) & \Delta y<0 \end{array} \end{array}$$

The results of the arcsine are always less than 90°, thus the real deviation angle *An* should be judged combining with Δ*x* and Δ*y*, which are the deviation between these two events center on the x and y direction, respectively.

**Figure 5:**
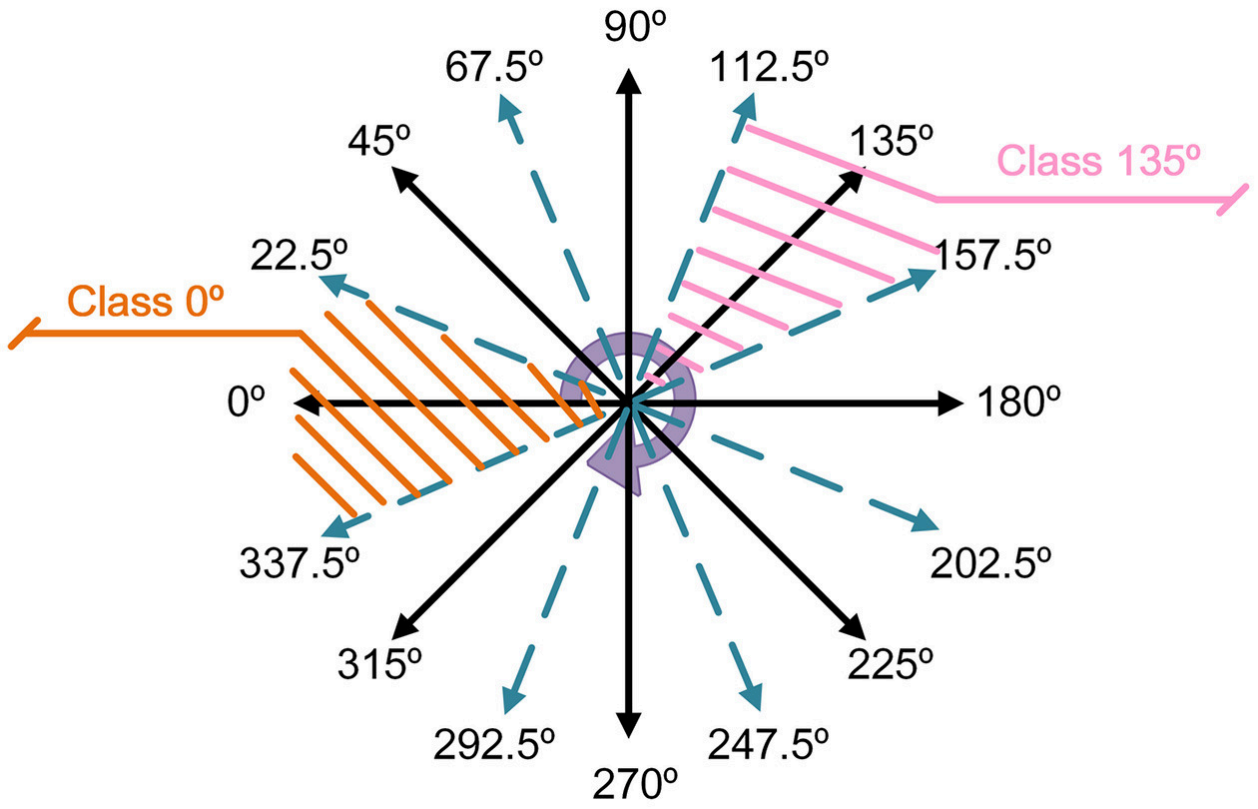
**The principle of orientation classification**.

In order to recognize objects moving in multiple orientations, the feature maps of testing samples need to be rotated back to 0° after feature extraction. To simplify the operation, 45° is chosen as a step. The orientation detector divides all angels into 8 classes (0°, 45°, 90°, 135°, 180°, 225°, 270°, 315°). If the angle belongs to a certain class within ±22.5°, it will be classified into the class of nearest angle. The detailed classification principle is summarized in Figure 5. After angel classification, the orientation detector sends the category label to the addresses LUT.

The aim of addresses LUT is finding out the corresponding relations of addresses between classified image and un-rotated image by rotation operation, and restoring the address information to the un-rotated image as much as possible. The mapped addresses of 8 classes are stored in the addresses LUT. These addresses are generated by the rotation matrix and the nearest neighbor interpolation, with which the relationship of position transformation can be figured out. The center of the input image is set as the origin of coordinates and the rotation angle is one of the 8 classified angles. As the maps in “C1” layer are rotated, the order of these maps has to be adjusted to make sure the orientation features match the corresponding features of training samples. The correspondence for each orientation feature is shown in Table 1. After the address transformation, the response spikes are conveyed to the neural network block. The neural network is trained in advance with samples in only reference orientation. With the operations described above, the features of the object moving in certain orientation are converted as that in reference orientation. Thus, the classifier can recognize it.

**Table 1:** **Orientation correspondence**.

**Class No**.	**Angle range**	**0°**	**45°**	**90°**	**135°**
1	−22.5° ~ 22.5°	0°	45°	90°	135°
2	22.5° ~ 67.5°	45°	90°	135°	0°
3	67.5° ~ 112.5°	90°	135°	0°	45°
4	112.5° ~ 157.5°	135°	0°	45°	90°
5	157.5° ~ 202.5°	0°	45°	90°	135°
6	202.5° ~ 247.5°	45°	90°	135°	0°
7	247.5° ~ 292.5°	90°	135°	0°	45°
8	292.5° ~ 337.5°	135°	0°	45°	90°

## Results

### On MNIST image dataset

#### Input generation

**Figure 6:**
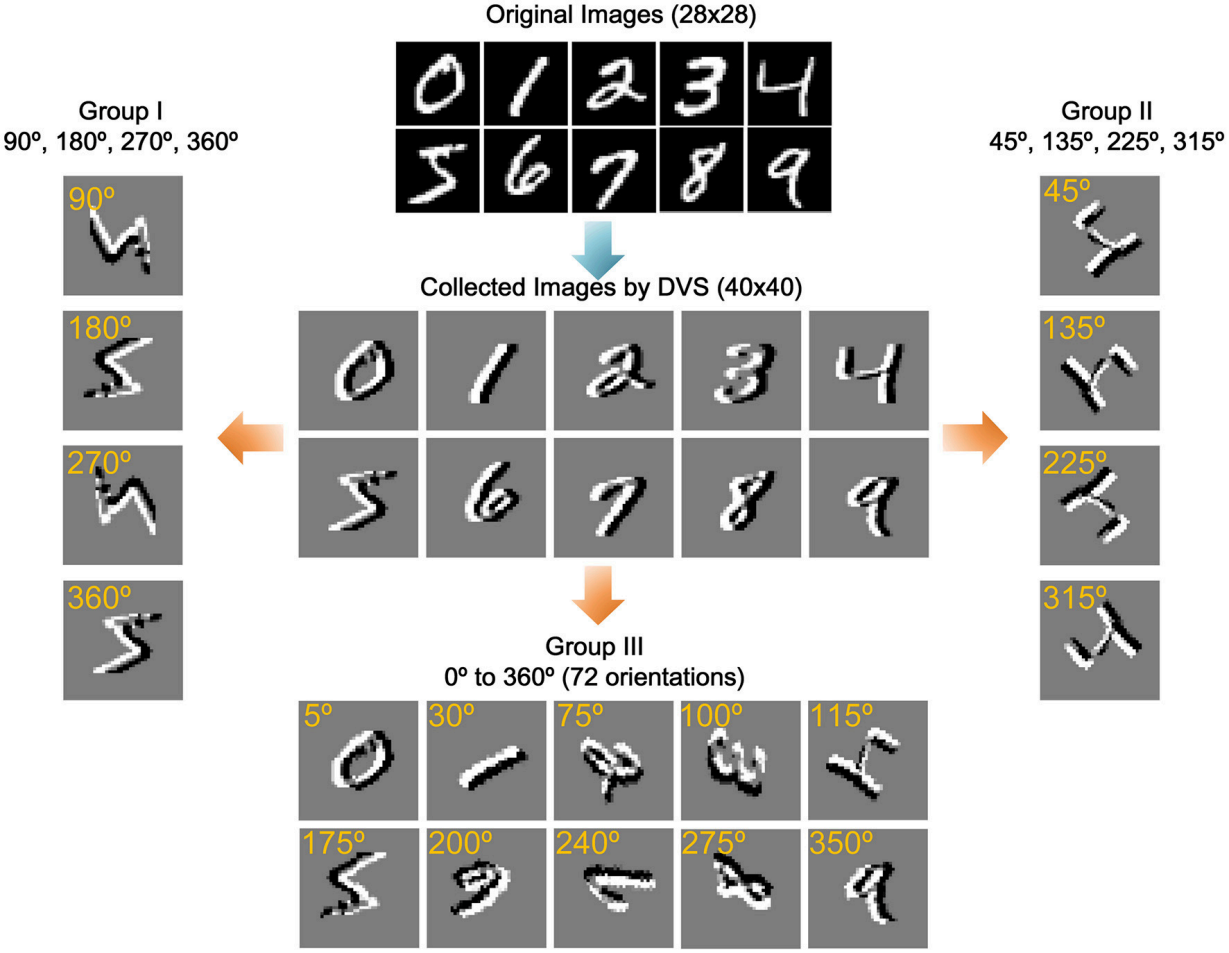
**Training samples and three groups of rotated samples**. The original images adopt a standard handwritten digit dataset from the Mixed National Institute of Standards and Technology (MNIST). The collected images simulate the moving state of objects by a differencing method. The remaining three groups of images are obtained by rotating the collected images in specific angle.

The multiple orientation event-based recognition system was implemented in MATLAB. In order to investigate the system performance, it is tested against a standard handwritten digit dataset from the Mixed National Institute of Standards and Technology (MNIST; Lecun et al., 1998) containing 10 digits (0–9) illustrated at the top of Figure 6. Note that the digits' order has been disrupted to ensure the randomness of input samples. To simulate the moving state of objects, we use a differencing method to form a new image just as shown in the center of Figure 6. In the differencing method, each column minuses another column with a certain interval. In this experiment, the interval is set as three. For example, the gray value of the first column is the difference between the fourth column and the first column. After the gray values of all columns are altered, a new image has been formed. In the meantime, the new image is converted to events via two encoding methods, in which the gray values map to one event or multiple events, respectively.

**Figure 7:**
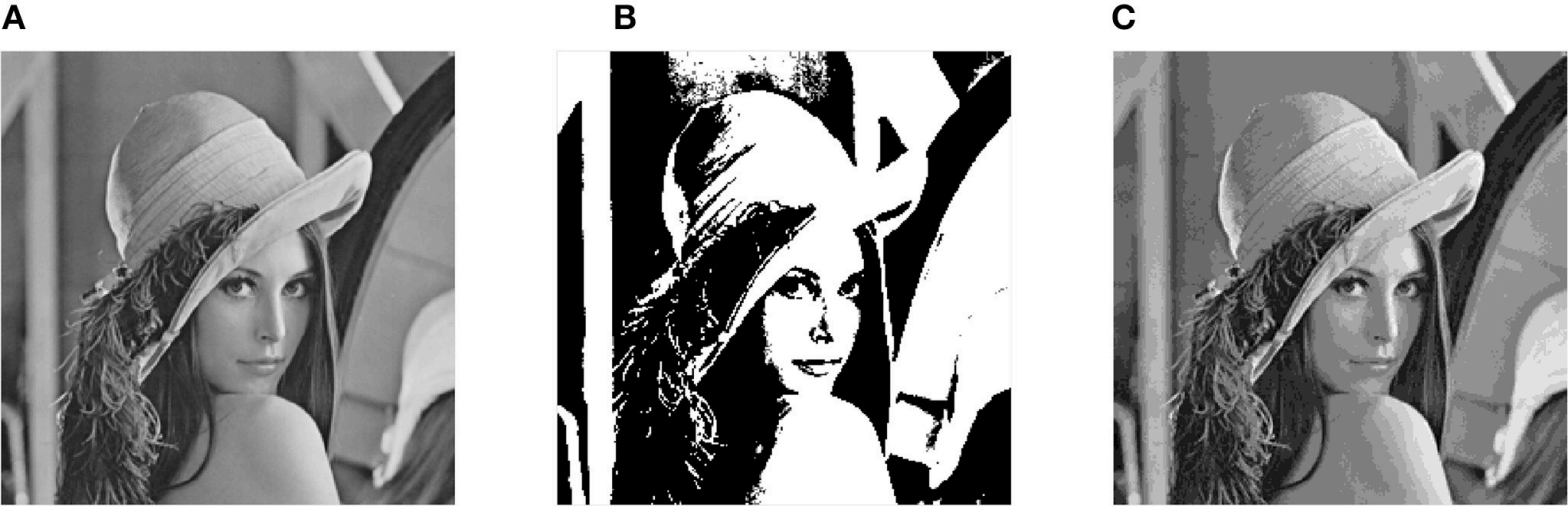
**Restored picture in different encoding methods. (A)** Original image. **(B)** One pixel one event encoding. **(C)** One pixel multiple event encoding.

The first encoding method compares each pixel with a threshold, and labels it with a positive (ON) or negative (OFF) polarity according to the comparison result. Thus, each pixel only generates one event. In fact, due to the difference of the light intensity change in each pixel, the number of events generated by different pixels may vary. In the second method, each pixel is assigned a number of events between 0 and 16 depending on its gray level, which means that 16 events correspond pixel gray value of 255. The events generated by two encoding methods are restored to images in Figure 7.

The original size of a sample image is 28 × 28. Due to the influence of rotation, the 28 × 28 image is enlarged to be a 40 × 40 image when it is rotated 45° which leads to the greatest difference in image size among all rotations. To keep all the images with identical size, all of the images are transferred from 28 × 28 to 40 × 40 by only adding 0 at four sides as background. The MNIST dataset possesses 70,000 pictures. In this experiment, 60,000 pictures were randomly selected as the training set and the rest 10,000 pictures are used as the testing set. The threshold operation in “C1” layer is set as 0.1 through repeated experiments. The membrane time constant τ_m_ and the leakage rate in the event-driven convolution is set as 1/τ_m_ = 5 × 10^4^ s^−1^. The max epoch of training is set as 10.

#### Results of MNIST dataset moving in specified orientation

In the training and testing experiment without rotation, the event-based recognition system with the first encoding method (one pixel one event) achieves an accuracy of 99.10% for the training set and 92.88% for the testing set. With the second encoding method (one pixel multiple events), the accuracy is 99.41% for the training set and 93.78% for the testing set. All the results are summarized in Table 2. As more events are generated for the moving object, the second encoding method possesses higher accuracy. The performance of the proposed system is compared with an original feedforward categorization system (Zhao et al., 2015). The difference between two systems is the use of the polarity information of events. The original feedforward categorization system presented by Zhao only deals with one polarity events which generate the silhouette of detected objects when reconstruct frames. In this paper, there are two polarities of events to describe the motion of objects. Compared with Zhao's method, the performance of the proposed system is similar or even better.

**Table 2:** **Accuracy of single orientation object recognition**.

**Algorithms**	**This work**		**Zhao's**
**Encoding**	**1–1**	**1–16**	**1–1**
training	99.10%	99.41%	99.36%
testing	92.88%	93.78%	91.29%

#### Results of MNIST dataset moving in multiple orientations

For further estimating the performance of the proposed system, three groups of rotated sample have been introduced for testing. In the first group, the 60,000 training samples and 10,000 testing samples are rotated in 4 standard orientations (90°, 180°, 270°, and 360°) and using a rotation matrix with the nearest neighbor interpolation in MATLAB as the new testing samples. Each sample is randomly rotated only once with one of the four degrees. In addition, the sample size remains 40 × 40 to keep the input of scale invariance. The second group of images are rotated in another 4 standard orientations (45°, 135°, 225°, 315°), and the third group of images are rotated randomly from 0 to 360° with a step of 5°, which is totally 72 orientations. Figure 6 shows some samples of data in three groups.

All these experiments are performed on a server with Intel Xeon X5670 and 64 GB physical RAM. In the first encoding method (one pixel one event), feature extraction time and orientation detection time are about 0.26 s and 0.042 s with 170 events. In the second encoding method, they are 2.5 s and 0.35 s with 1676 events. The time of orientation detection is always lower than feature extraction time, which indicates that orientation detection can be completed before feature extraction while running in parallel. Therefore, the parallel architecture that orientation detector works synchronously with the feature extractor, can be realized without any delay in the classification.

**Figure 8:**
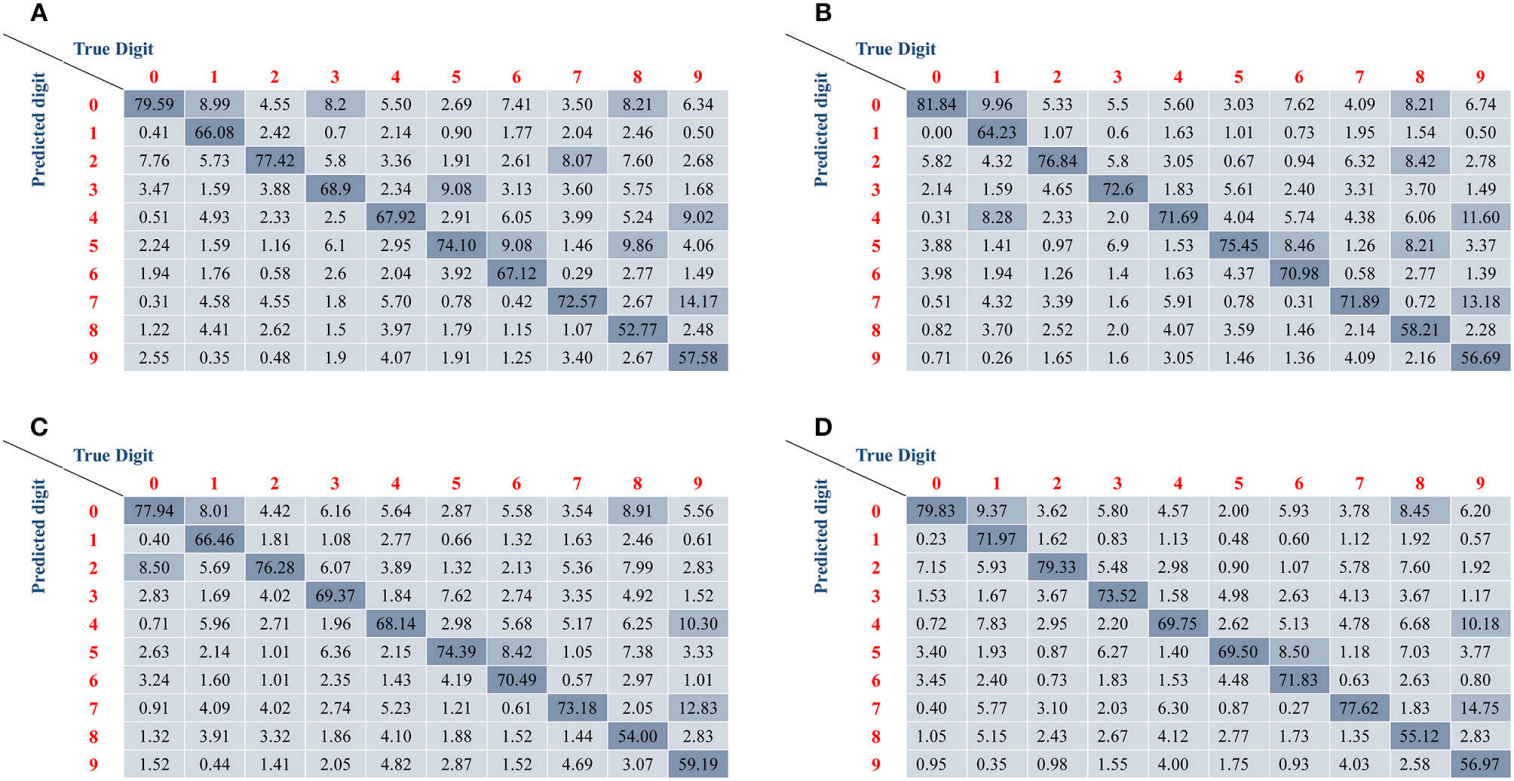
**The confusion matrices of different samples**. The resulting confusion matrices from training samples with **(A)** one pixel one event encoding and **(B)** one pixel multiple events encoding. The resulting confusion matrix from testing samples with **(C)** one pixel one event encoding and **(D)** one pixel multiple events encoding. All results show higher accuracy for simple digits (such as 0, 1, 2, and 7) and lower accuracy for difficult digits (such as 8 and 9).

All the accuracies of recognition and orientation detection are listed in Table 3. The results show that the accuracy of orientation detection in the first two groups is over 99% or even achieves 100%, but the accuracy of the last group is only more than 90 and 95% in different encoding method, respectively. The reduction of recognition ratio is because some angles are difficult to define when they lie in between two adjacent angle classes. In terms of classification, The Group I has high accuracy and is almost the same as the accuracy of un-rotated samples. However, it is obvious that the Group II and the Group III have lower accuracy ranging from 67.98 to 70.54%. Although Group II has a low accuracy, the basic classification function can be realized. The Group III imitates free angle pattern, and the accuracy is between Group I and Group III. Similar with the un-rotated experiment, the second encoding method (one pixel multiple events) has higher accuracy than the first encoding method (one pixel one event) in all rotated angles. Figure 8 illustrates the confusion matrices of all the digits in different processing methods. The confusion matrices show the classification distribution. The values in the tables are the corresponding probability that the actual digit (column-wise) is identified as the digits as represented row-wise. From the results, the digits with simple shape “0,” “1,” “2,” and “7” can attain good accuracy, whereas the more complex digits like “8” and “9” exhibit lower accuracy. For example, “9” is mostly misclassified as “4” and “7,” since they have similar features.

**Table 3:** **Accuracy of multiple orientation object recognition**.

**Class No**.	**Group I** **(90°, 180°, 270°, 360°)**		**Group II** **(45°, 135°, 225°, 315°)**		**Group III** **(72 orientations)**	
**Encoding**	**1–1**	**1–16**	**1–1**	**1–16**	**1–1**	**1–16**
**(A) ACCURACY OF ORIENTATION**						
Training	99.96%	100%	99.58%	99.95%	90.47%	95.20%
Testing	99.95%	100%	99.44%	99.90%	90.70%	95.31%
**(B) ACCURACY OF CLASSIFICATION**						
Training	97.81%	99.19%	68.21%	70.35%	68.95%	70.54%
Testing	92.71%	93.68%	67.98%	69.75%	68.37%	69.92%

#### Results of MNIST dataset collected by DVS

**Figure 9:**
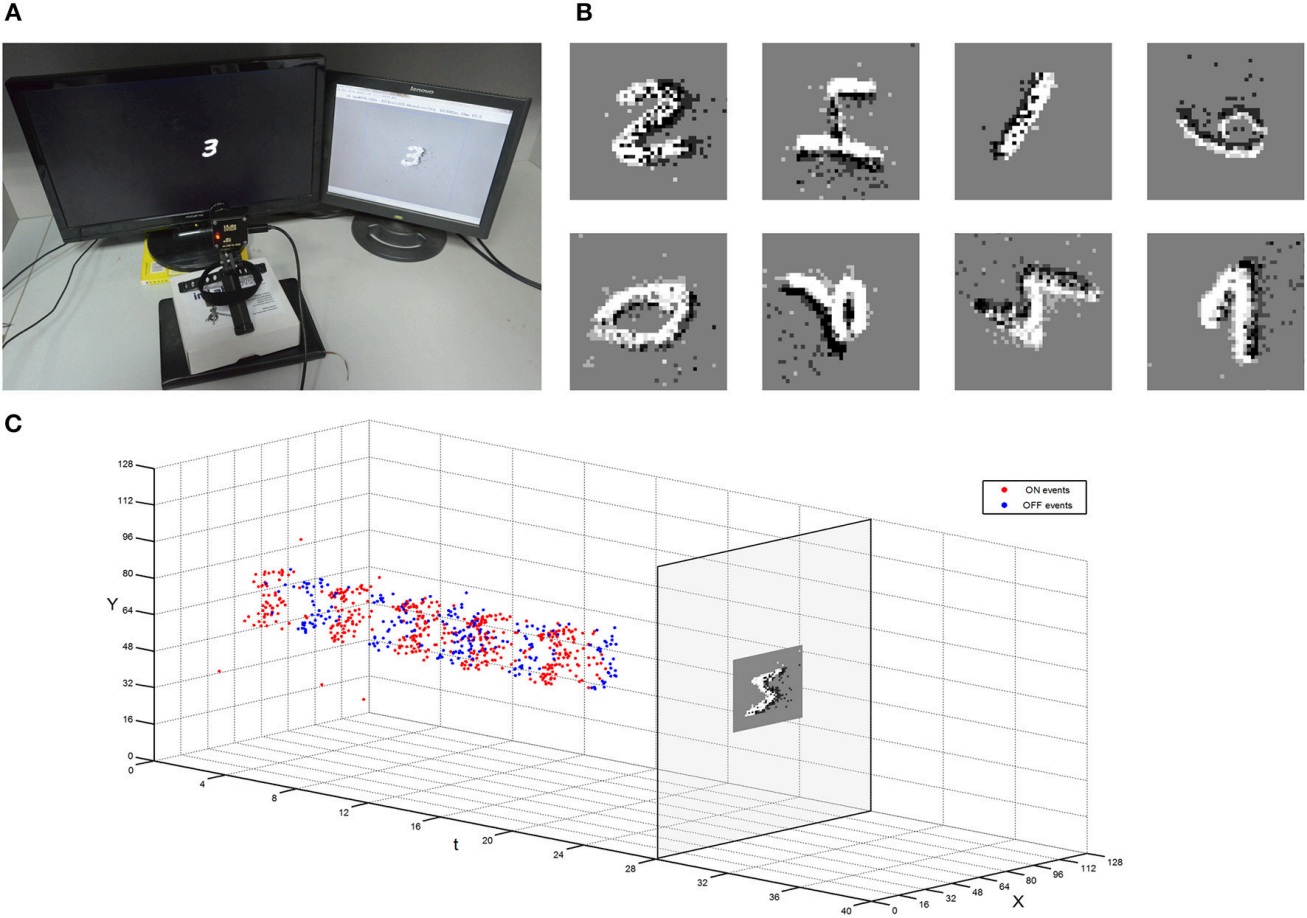
**Experiment setup of capturing the moving MNIST dataset by DVS. (A)** A DVS records the moving digit on an LCD monitor. **(B)** Example of different moving MNIST digits captured with DVS. **(C)** The procedure of the restoration from the collected events to a snapshot.

The MNIST dataset is also collected using a dynamic vision sensor whose input space is 128 × 128 pixels (Lichtsteiner et al., 2008). The data acquisition method draws lessons from the MNIST-DVS (Serrano-Gotarredona and Linares-Barranco, 2015b) that an LCD monitor displays moving digits. The testing digits are the original MNIST digit 28 × 28 pixel pictures moving at constant speed from the right edge of the monitor to the left (about 2 s). The monitor's frame frequency is set to 75 Hz, which is the maximum possible value to reduce artifacts. The distance from DVS to monitor is fixed to capture digits in 40 × 40 which is the same as the training samples. The captured events are converted into a video at 30 fps by jAERViewer software (Delbrück, 2006), and it is displayed on another LCD. The experiment setup and procedure were shown in Figure 9A. The digits moving in multiple orientations are acquired as testing samples which are shown in Figure 9B. The event flow of one sample is illustrated in Figure 9C. All the testing samples in Figure 9B could be recognized in this system trained with MNIST samples only moving in the single referencev orientation.

### On poker-DVS dataset

**Figure 10:**
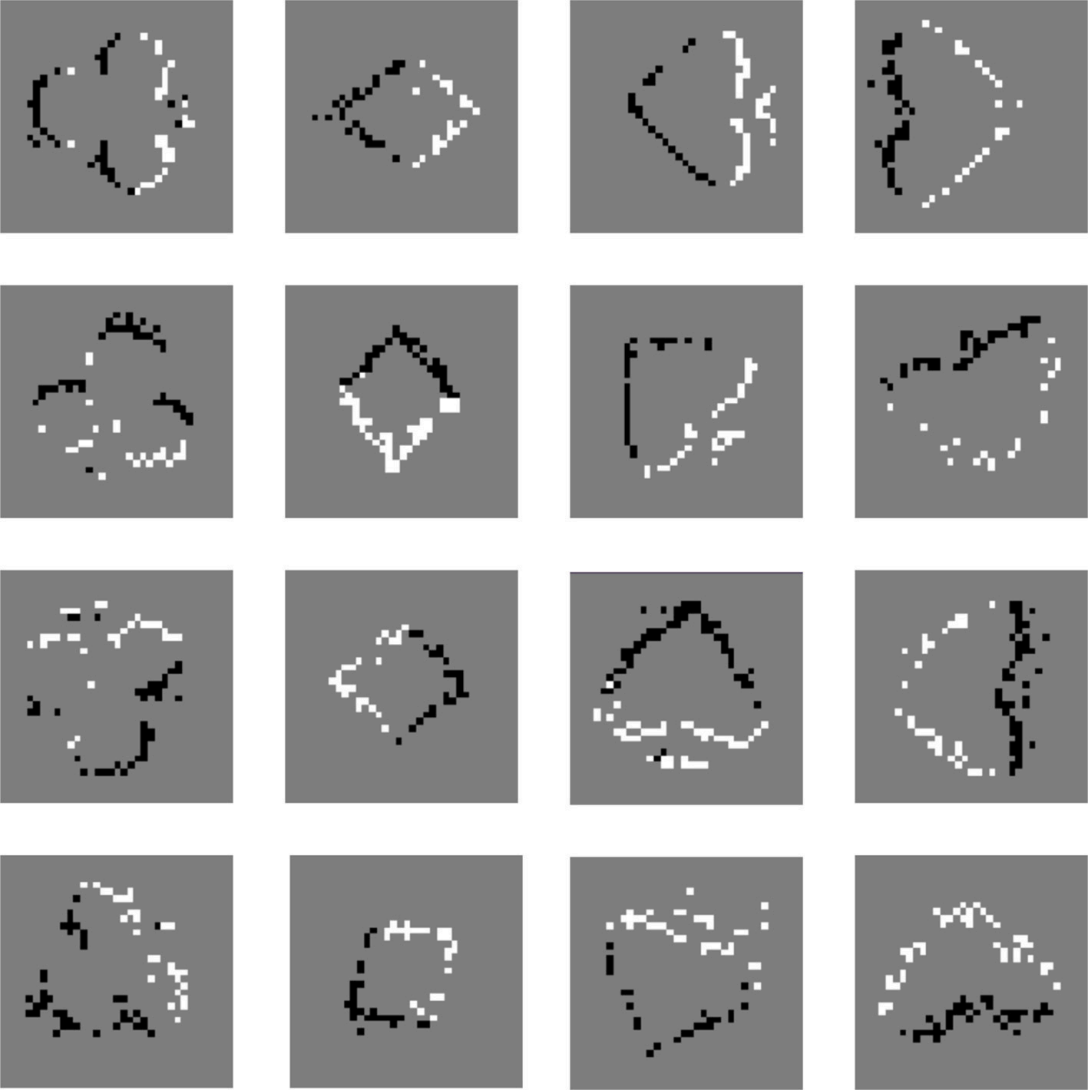
**Poker dataset with DVS mapped as snapshots**. The snapshots in the first row are restored from the training samples, and the rest images are made from testing samples.

The poker dataset provided by Serrano-Gotarredona and Linares-Barranco (2015a) consists of 4 card types (spades, hearts, diamonds, and clubs), and it is captured by a DVS. The poker dataset is made by fast browsing of a poker deck with all the pips in black. The training samples are prepared by intercepting about 1200 events in a 40 × 40 array for each pattern. The moving direction of poker dataset is along the positive direction of the y axis. Then the addresses of poker dataset were altered to rotate the initial positive orientation to the reverse direction of the x axis as that of the MNIST dataset. Samples of the four types were rotated from 0° to 360° with a step of 5° as the testing samples. There are 864 testing samples in total, and each pip has 3 samples in each orientation. The images in the first row of Figure 10 are restored from the training samples, and the rest images are made from testing samples.

In the experiments, 864 testing samples were tested several times with the system trained with 100–800 samples. This system classified card pips with an accuracy of 59.95% with 100 training samples. When the number of training samples reached 800, the accuracy of recognition rose to 66.09%. Furthermore, another experiment was done with additional 200 samples, whose orientations are less than ±20°. With the better trained system, the accuracy of recognition approached to 76.39%. All these results are summarized in Table 4. The results suggest that using samples with slightly varied orientations can improve performance in term of recognition accuracy.

**Table 4:** **Accuracy of poker pattern recognition**.

**Training sample size**	**100**	**500**	**800**	**800+200**
Accuracy	59.95%	64.00%	66.09%	76.39%

### On vehicle-DVS dataset

**Figure 11:**
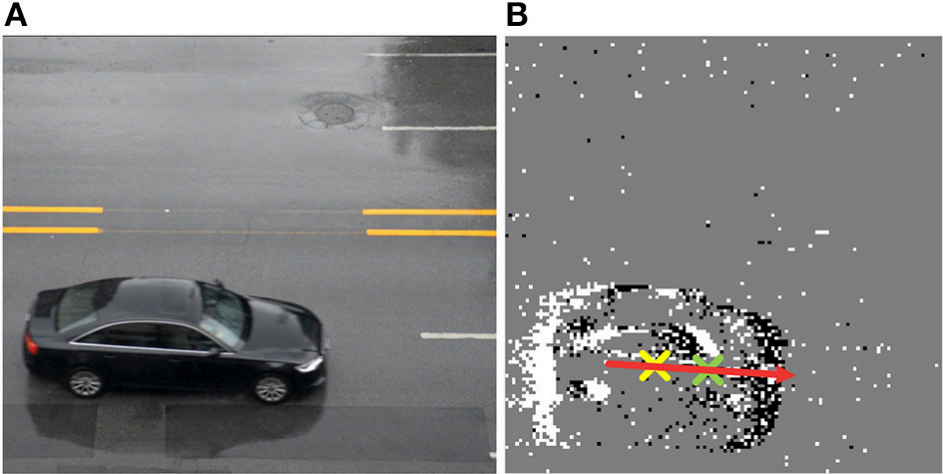
**The orientation detection of moving vehicle collected by DVS in real time. (A)** Picture taken with a frame-driven camera. **(B)** Snapshot mapping by AER events and the arrow shows the orientation of a moving vehicle.

This system is suitable for traffic surveillance application, yet it is too heavy to extract a large amount of formatted vehicle samples. Therefore, only the Gaussian tracker's accuracy for position and orientation are evaluated. A dynamic vision sensor with 128 × 128 pixels is used to capture the vehicle moving in different orientations. As an example, AER data with 2894 events during the time interval about 39,956 ns are transmitted into the orientation detector of this system. A comparison between a real time picture captured by a frame-driven camera and a snapshot mapped by AER events can be found in Figure 11. The centers of ON and OFF events are figured out by the orientation detector are marked with crosses in red and blue, respectively, in Figure 11B. Furthermore, the result of moving orientation is presented as an arrow in the snapshots and deviation angle is 184.6°, which is very close to the deviation angle 182.1° measured from the real time picture. This error may not completely come from the orientation detector, but the position difference of the two cameras may also contribute to errors. Although the noises are introduced in the acquired AER data from the DVS, the orientation detection still has good performance in orientation detection.

## Discussion

This paper presents a multiple orientation event-based recognition system used for asynchronous AER vision sensors. In the system, a hierarchical architecture of bio-inspired feature extraction block extracts multiple orientations and scales feature. A network of LIF neurons that flexibly simulates mammalian brain classifies the objects according their features. In this paper, an orientation detector is added to track the orientation of moving objects. The tracking works with feature extracting simultaneously, without any increase in classification time. In this event-driven system, two polarity events (ON events and OFF events) are introduced to achieve higher identification and detect moving orientation. The algorithm of orientation detection and address restoration are presented in this paper. It makes multi-orientation event-based recognition possible by training dataset only with certain reference positive orientation. This system equips a high potential for innovation and development in architecture, static datasets making, and multi-orientation recognition function.

Various kinds of objects were tested with this system, such as MNIST, pokers, and vehicles. For the MNIST dataset, to simulate the moving state of objects, a differencing method is applied to generate a new image dataset. The AER dataset mapped by image dataset could be used to classify data acquired by a real DVS, which shortens the time in gathering training samples one by one. As the results illustrate, our system performs well with both samples converted from static images and collected by DVS.

However, this system is not completely free of problems: One typical problem is orientation classification. It classifies angles into 8 classes, which increases the accuracy of orientation detection, but it also has a negative effect on recognition. The fault tolerance of 8 angle classes is good when the center of ON and OFF events are very close such as highly patterned objects. However, the error between the actual angle and the classified angle may lead to mistakes in recognition. Therefore, in the experiment of pokers, additional training samples with slightly deviated orientations less than ±20° were added to enhance the accuracy of recognition. A further problem is the recognition result is only close to 70%. As mentioned in the MNIST experimental results, the group of images rotated in 4 standard orientations (45°, 135°, 225°, 315°) has lower accuracy ranging from 67.98 to 70.35%. The primary reasons may be summarized as follows: the extracting strength of the Gabor filter between 45° and 0° features differs a lot, and the rotation of all the maps in “C1” layer leads to distortion for features.

This system can recognize objects with various orientations moving along its positive direction. With respect to objects moving in an arbitrary direction, this system may fail. Furthermore, it may also fail to recognize targets with different orientation displaying in a flashing way, due to the lack of ON and OFF events pairs. Therefore, the application field of this proposed method is limited in monitoring targets with various orientations that has a certain moving direction like vehicles, vessels, airplanes, and so on. Future work is to improve the recognition accuracy and extend the ability to recognize more kind of objects with various orientations and moving directions.

## Author contributions

Models/experiments design: HW, JX, and ZG. Mathematical and theoretical analysis: SY and JM. Experimental investigations: HW, ZG, and CL. Manuscript preparation: HW and ZG. All authors reviewed the manuscript.

### Conflict of interest statement

The authors declare that the research was conducted in the absence of any commercial or financial relationships that could be construed as a potential conflict of interest.

## References

[B1] Aung Myat Thu L. Do Anh T. Chen S. Yeo K. S. (2011). Adaptive priority toggle asynchronous tree arbiter for AER-based image sensor, in 2011 IEEE/IFIP 19th International Conference on VLSI and System-on-Chip (Kowloon), 66–71.

[B2] Boahen K. A. (2000). Point-to-point connectivity between neuromorphic chips using address events. IEEE Trans. Circ. Syst. II Analog Digit. Signal Process. 47, 416–434. 10.1109/82.842110

[B3] Brandli C. Berner R. Yang M. Liu S.-C. Delbruck T. (2014). A 240 × 180 130 db 3 μs latency global shutter spatiotemporal vision sensor. IEEE J. Solid State Circ. 49, 2333–2341. 10.1109/JSSC.2014.2342715

[B4] Chen L. P. Lu G. J. Zhang D. S. (2004). Effects of different Gabor Filter parameters on image retrieval by texture, in Multimedia Modelling Conference (Brisbane, QLD), 273–278.

[B5] Chen S. Akselrod P. Zhao B. Carrasco J. A. P. Linares-Barranco B. Culurciello E. (2012). Efficient feedforward categorization of objects and human postures with address-event image sensors. IEEE Trans. Pattern Anal. Mach. Intell. 34, 302–314. 10.1109/TPAMI.2011.120 21670481

[B6] Cohen G. K. Orchard G. Leng S. H. Tapson J. Benosman R. B. van Schaik A. (2016). Skimming digits: neuromorphic classification of spike-encoded images. *Front*. Neurosci. 10:184 10.3389/fnins.2016.00184 PMC4848313 27199646

[B7] Culurciello E. Etienne-Cummings R. Boahen K. A. (2003). A biomorphic digital image sensor. IEEE J. Solid State Circ. 38, 281–294. 10.1109/JSSC.2002.807412

[B8] Delbrück T. (2006). Available online at: http://sourceforge.net/projects/jaer/

[B9] Delbrück T. Lichtsteiner P. (2007). Fast sensory motor control based on event-based hybrid neuromorphic-procedural system, in 2007 IEEE International Symposium on Circuits and Systems (New Orleans, LA), 845–848.

[B10] Drazen D. Lichtsteiner P. Häfliger P. Delbrück T. Jensen A. (2011). Toward real-time particle tracking using an event-based dynamic vision sensor. Exp. Fluids 51, 1465–1469. 10.1007/s00348-011-1207-y

[B11] Fukushima K. (1980). Neocognitron: a self-organizing neural network model for a mechanism of pattern recognition unaffected by shift in position. Biol. Cybern. 36, 193–202. 10.1007/BF00344251 7370364

[B12] Han S. Feng-Gang H. (2005). Human gait recognition based on motion analysis, in 2005 International Conference on Machine Learning and Cybernetics (Guangzhou), 4464–4468.

[B13] Lagorce X. Meyer C. Ieng S. H. Filliat D. Benosman R. (2015). Asynchronous event-based multikernel algorithm for high-speed visual features tracking. IEEE Trans. Neural Netw. Learn. Syst. 26, 1710–1720. 10.1109/TNNLS.2014.2352401 25248193

[B14] Lecun Y. Bottou L. Bengio Y. Haffner P. (1998). Gradient-based learning applied to document recognition. Proc. IEEE 86, 2278–2324. 10.1109/5.726791

[B15] Lichtsteiner P. Posch C. Delbrück T. (2008). A 128 × 128 120dB 15μs latency asynchronous temporal contrast vision sensor. IEEE J. Solid State Circ. 43, 566–576. 10.1109/JSSC.2007.914337

[B16] Litzenberger M. Kohn B. Belbachir A. N. Donath N. Gritsch G. Garn H. (2006a). Estimation of vehicle speed based on asynchronous data from a silicon retina optical sensor, in 2006 IEEE Intelligent Transportation Systems Conference (Toronto, ON), 653–658.

[B17] Litzenberger M. Posch C. Bauer D. Belbachir A. N. Schon P. Kohn B. (2006b). Embedded vision system for real-time object tracking using an asynchronous transient vision sensor, in 2006 IEEE 12th Digital Signal Processing Workshop & 4th IEEE Signal Processing Education Workshop (Los Alamitos, CA: IEEE), 173–178.

[B18] Mahowald M. A. Mead C. (1991). The silicon retina. Sci. Am. 264, 76–82. 10.1038/scientificamerican0591-76 2052936

[B19] Markram H. Lübke J. Frotscher M. Sakmann B. (1997). Regulation of synaptic efficacy by coincidence of postsynaptic APs and EPSPs. Science 275, 213–215. 10.1126/science.275.5297.213 8985014

[B20] Ni Z. Pacoret C. Benosman R. Ieng S. Régnier S. (2012). Asynchronous event-based high speed vision for microparticle tracking. J. Microsc. 245, 236–244. 10.1111/j.1365-2818.2011.03565.x

[B21] Orchard G. Jayawant A. Cohen G. K. Thakor N. (2015a). Converting static image datasets to spiking neuromorphic datasets using saccades. Front. Neurosci. 9:437. 10.3389/fnins.2015.00437 26635513 PMC4644806

[B22] Orchard G. Meyer C. Etienne-Cummings R. Posch C. Thakor N. Benosman R. (2015b). HFirst: a temporal approach to object recognition. IEEE Trans. Pattern Anal. Mach. Intell. 37, 2028–2040. 10.1109/TPAMI.2015.2392947 26353184

[B23] Pérez-carrasco J. A. Zhao B. Serrano C. Acha B. Serrano-Gotarredona T. Chen S.. (2013). Mapping from frame-driven to frame-free event-driven vision systems by low-rate rate coding and coincidence processing–Application to feedforward ConvNets. IEEE Trans. Pattern Anal. Mach. Intell. 35, 2706–2719. 10.1109/TPAMI.2013.71 24051730

[B24] Posch C. Matolin D. Wohlgenannt R. (2008). An asynchronous time-based image sensor, in 2008 IEEE International Symposium on Circuits and Systems (ISCAS) (Washington, DC), 2130–2133.

[B25] Posch C. Matolin D. Wohlgenannt R. (2011). A QVGA 143dB dynamic range frame-free PWM image sensor with lossless pixel-level video compression and time-domain CDS. IEEE J. Solid State Circ. 46, 259–275. 10.1109/JSSC.2010.2085952

[B26] Riesenhuber M. Poggio T. (1999). Hierarchical models of object recognition in cortex. Nat. Neurosci. 2, 1019–1025. 10.1038/14819 10526343

[B27] Serrano-Gotarredona R. Oster M. Lichtsteiner P. Linares-Barranco A. Paz-Vicente R. Gomez-Rodriguez F.. (2009). CAVIAR: a 45k neuron, 5M synapse, 12G connects/s AER hardware sensory-processing-learning-actuating system for high-speed visual object recognition and tracking. IEEE Trans. Neural Netw. 20, 1417–1438. 10.1109/TNN.2009.2023653 19635693

[B28] Serrano-Gotarredona R. Serrano-Gotarredona T. Acosta-Jimenez A. Serrano-Gotarredona C. Perez-Carrasco J. A. Linares-Barranco B. (2008). On real-time AER 2-D convolutions hardware for neuromorphic spike-based cortical processing. IEEE Trans. Neural Netw. 19, 1196–1219. 10.1109/TNN.2008.2000163

[B29] Serrano-Gotarredona T. Linares-Barranco B. (2013). A 128 × 128 1.5% contrast sensitivity 0.9% FPN 3μs latency 4mW asynchronous frame-free dynamic vision sensor using transimpedance preamplifiers. IEEE J. Solid State Circ. 48, 827–838. 10.1109/JSSC.2012.2230553

[B30] Serrano-Gotarredona T. Linares-Barranco B. (2015a). Available online at: http://www2.imse-cnm.csic.es/caviar/POKERDVS.html

[B31] Serrano-Gotarredona T. Linares-Barranco B. (2015b). Poker-DVS and MNIST-DVS. Their history, how they were made, and other details. Front. Neurosci. 9:481. 10.3389/fnins.2015.00481 26733794 PMC4686704

[B32] Serre T. (2006). Learning a Dictionary of Shape-Components in Visual Cortex: Comparison with Neurons, Humans and Machines. Ph.D. dissertation. Massachusetts Institute of Technology, Cambridge, MA.

[B33] Serre T. Wolf L. Bileschi S. Riesenhuber M. Poggio T. (2007). Robust object recognition with cortex-like mechanisms. IEEE Trans. Pattern Anal. Mach. Intell. 29, 411–426. 10.1109/TPAMI.2007.56 17224612

[B34] Triesch J. Malsburg C. V. D. (2001). A system for person-independent hand posture recognition against complex backgrounds. IEEE Trans. Pattern Anal. Mach. Intell. 23, 1449–1453. 10.1109/34.977568

[B35] Tsitiridis A. Conde C. Diego I. M. D. Saez J. S. D. R. Gomez J. R. Cabello E. (2015). Gabor feature processing in spiking neural networks from retina-inspired data, in 2015 International Joint Conference on Neural Networks (IJCNN) (Killarney), 1–8.

[B36] Valeiras D. R. Lagorce X. Clady X. Bartolozzi C. Ieng S. H. Benosman R. (2015). An asynchronous neuromorphic event-driven visual part-based shape tracking. IEEE Trans. Neural Netw. Learn. Syst. 26, 3045–3059. 10.1109/TNNLS.2015.2401834 25794399

[B37] Wulfram G. Werner M. K. (2002). Spiking neuron models: single neurons, populations, plasticity. Encyclopedia Neurosci. 4, 277–280. 10.1017/CBO9780511815706

[B38] Yu Q. Tang H. Tan K. C. Li H. (2013). Rapid feedforward computation by temporal encoding and learning with spiking neurons. IEEE Trans. Neural Netw. Learn. Syst. 24, 1539–1552. 10.1109/TNNLS.2013.2245677 24808592

[B39] Zaghloul K. A. Boahen K. (2004). Optic nerve signals in a neuromorphic chip I: outer and inner retina models. IEEE Trans. Biomed. Eng. 51, 657–666. 10.1109/TBME.2003.821039 15072220

[B40] Zhao B. Chen S. (2011). Realtime feature extraction using MAX-like convolutional network for human posture recognition, in 2011 IEEE International Symposium of Circuits and Systems (ISCAS) (Rio de Janeiro), 2673–2676.

[B41] Zhao B. Ding R. Chen S. Linares-Barranco B. Tang H. (2015). Feedforward categorization on AER motion events using cortex-like features in a spiking neural network. IEEE Trans. Neural Netw. Learn. Syst. 26, 1963–1978. 10.1109/TNNLS.2014.2362542 25347889

